# Variable Genomic Landscapes of Advanced Melanomas with Heavy Pigmentation

**DOI:** 10.1093/oncolo/oyac090

**Published:** 2022-05-12

**Authors:** Richard S P Huang, Julie Y Tse, Lukas Harries, Ryon P Graf, Douglas I Lin, Karthikeyan Murugesan, Matthew C Hiemenz, Vamsi Parimi, Tyler Janovitz, Brennan Decker, Eric Severson, Mia A Levy, Shakti H Ramkissoon, Julia A Elvin, Jeffrey S Ross, Erik A Williams

**Affiliations:** Foundation Medicine, Inc., Cambridge, MA, USA; Foundation Medicine, Inc., Cambridge, MA, USA; Foundation Medicine, Inc., Cambridge, MA, USA; Foundation Medicine, Inc., Cambridge, MA, USA; Foundation Medicine, Inc., Cambridge, MA, USA; Foundation Medicine, Inc., Cambridge, MA, USA; Foundation Medicine, Inc., Cambridge, MA, USA; Foundation Medicine, Inc., Cambridge, MA, USA; Foundation Medicine, Inc., Cambridge, MA, USA; Foundation Medicine, Inc., Cambridge, MA, USA; Foundation Medicine, Inc., Cambridge, MA, USA; Foundation Medicine, Inc., Cambridge, MA, USA; Rush University Medical Center, Chicago, IL, USA; Foundation Medicine, Inc., Cambridge, MA, USA; Wake Forest Comprehensive Cancer Center, and Department of Pathology, Wake Forest School of Medicine, Winston-Salem, NC, USA; Foundation Medicine, Inc., Cambridge, MA, USA; Foundation Medicine, Inc., Cambridge, MA, USA; Department of Pathology, State University of New York (SUNY) Upstate Medical University, Syracuse, NY, USA; Foundation Medicine, Inc., Cambridge, MA, USA; Department of Pathology, Department of Dermatology, UCSF Dermatopathology Service, University of California San Francisco, San Francisco, CA, USA; Department of Pathology and Laboratory Medicine, University of Miami, Sylvester Comprehensive Cancer Center, and Jackson Memorial Hospitals, Miami, FL, USA

**Keywords:** heavily pigmented, melanomas, PD-L1 immunohistochemistry, comprehensive genomic profiling, biomarkers

## Abstract

**Background:**

In the current study, we examined the real-world prevalence of highly pigmented advanced melanomas (HPMel) and the clinicopathologic, genomic, and ICPI biomarker signatures of this class of tumors.

**Materials and Methods:**

Our case archive of clinical melanoma samples for which the ordering physician requested testing for both PD-L1 immunohistochemistry (IHC) and comprehensive genomic profiling (CGP) was screened for HPMel cases, as well as for non-pigmented or lightly pigmented advanced melanoma cases (LPMel).

**Results:**

Of the 1268 consecutive melanoma biopsies in our archive that had been submitted for PD-L1 IHC, 13.0% (165/1268) were HPMel and 87.0% (1103/1268) were LPMel. In the HPMel cohort, we saw a significantly lower tumor mutational burden (TMB, median 8.8 mutations/Mb) than in the LPMel group (11.4 mut/Mb), although there was substantial overlap. In examining characteristic secondary genomic alterations (GA), we found that the frequencies of GA in *TERTp*, *CDKN2A*, *TP53*, and *PTEN* were significantly lower in the HPMel cases than in LPMel. A higher rate of GA in *CTNNB1*, *APC*, *PRKAR1A*, and *KIT* was identified in the HPMel cohort compared with LPMel.

**Conclusions:**

In this study, we quantified the failure rates of melanoma samples for PD-L1 testing due to high melanin pigmentation and showed that CGP can be used in these patients to identify biomarkers that can guide treatment decisions for HPMel patients. Using this practical clinical definition for tumor pigmentation, our results indicate that HPMel are frequent at 13% of melanoma samples, and in general appear molecularly less developed, with a lower TMB and less frequent secondary GA of melanoma progression.

## Teaser

Despite the importance of immune checkpoint inhibitors (ICPI) in the treatment of melanoma, current methods for PDL1 immunohistochemistry (IHC) do not allow interpretation when the tumor contains abundant melanin that obscures the IHC stain. This article examines the real-world prevalence of highly pigmented advanced melanomas and the clinicopathologic, genomic, and ICPI biomarker signatures of this class of tumors.

**Implications for Practice**

In this study, we quantified the failure rates of melanoma samples for PD-L1 testing due to high melanin pigmentation and showed that CGP can be used in these patients to identify biomarkers that can guide treatment decisions for patients with HPMel. Using this practical clinical definition for tumor pigmentation, our results indicate that HPMel are frequent at 13% of melanoma samples, and in general appear molecularly less developed, with a lower tumor mutational burden (TMB) and less frequent secondary GA of melanoma progression. However, TMB is highly overlapping between HPMel and LPMel, suggesting that HPMel would nevertheless benefit from ICPI.

## Introduction

Melanoma remains a deadly disease worldwide, with 106 110 new diagnoses and 7180 deaths estimated in the US in 2021.^1^ Surgical excision remains the main treatment for patients with localized melanoma. In clinically advanced disease, targeted therapies such as *BRAF* and *MEK* inhibitors are given for tumors that carry activating mutations in *BRAF.* More recently, monoclonal antibodies against cytotoxic T lymphocyte-associated-4 (CTLA-4), programmed death ligand-1 (PD-L1), and programmed death-1 (PD-1) have become widely used.^2^ The 2 PD-1 inhibitors, nivolumab and pembrolizumab, that have an indication for advanced melanoma from the US Food and Drug Administration (FDA) were both approved without companion diagnostics (CDx) to assess likely tumor responsiveness before initiation of ICPI. While the key clinical trials of these 2 agents showed tumor objective response rates (ORRs, 40% and 52%, respectively) and 5-year overall survival (OS, both 34%) that are superior to the benefits from standard systemic therapies for patients with melanoma, new biomarkers are needed to stratify patients with advanced melanoma into likely responders vs non-responders to ICPI.^3-6^

Currently, PD-L1 immunohistochemistry (IHC) is FDA-approved as a CDx for several ICPIs in specific non-melanoma tumor types.^7,8^ In 2 different pan-solid tumor approvals that include melanoma, tumor mutational burden (TMB) ≥10 mutations/Mb and microsatellite instability-high (MSI-High) were each approved as a separate CDx for pembrolizumab in patients that have exhausted standard of care options.^9,10^ In addition, evidence exists in the literature that *CD274* (encodes for PD-L1) amplifications and losses can predict responses to ICPI.^11-14^ Finally, GA in *PBRM1*, *STK11*, or *KEAP1*, amplifications of *MDM2/4*, and APOBEC mutational signature have also been suggested as predictive biomarkers for ICPI response in other tumor types.^15-22^

Despite the importance of ICPI in the treatment of advanced melanoma, current methods for PD-L1 IHC do not allow interpretation when the tumor contains abundant melanin that obscures the IHC stain. In the current study, we examined the real-world prevalence of highly pigmented advanced melanomas (HPMel), defined here by the presence of melanin pigmentation that rendered IHC uninterpretable. In addition, we examined the clinicopathologic, genomic, and ICPI biomarker signatures of this class of tumors. Non-pigmented or lightly pigmented advanced melanoma cases (LPMel), defined here by interpretable IHC for PD-L1, served as our comparator group.

## Materials and Methods

### Patients and Tumor Samples

Our overall archive of 367 651 tumor samples, each from a different patient, were sent from medical care facilities across North America for comprehensive genomic profiling (CGP) for detection of targetable GA during routine clinical care. We screened our archive for melanoma cases between January 2018 and May 2021 for which the ordering physician had also requested PD-L1 IHC assay (*n* = 1268 consecutive cases). Age and sex of patient and specimen site were extracted from accompanying pathology reports. Approval for this study was obtained from the Western Institutional Review Board Protocol No. 20152817, including issuing an informed consent waiver and a HIPAA waiver of authorization.

### PD-L1 IHC and Determination of Heavily Pigmented Melanomas vs Non/Lightly Pigmented Melanomas

All PD-L1 IHC testing were performed using the DAKO 22C3 PharmDx assay (Agilent, Santa Clara, CA) following the manufacturer’s instructions in a Clinical Laboratory Improvement Amendments (CLIA)-certified and College of American Pathologists (CAP)-accredited reference laboratory (Foundation Medicine, Morrisville, NC). A positive PD-L1 IHC produces a brown stain that can be obscured by heavy melanin pigmentation, rendering it impossible to distinguish positive from negative results. Whenever this technical problem resulted in uninterpretable IHC, it was documented, allowing us to identify HPMel cases. As noted above, cases with interpretable IHC were designated LPMel.

### Comprehensive Genomic Profiling

Comprehensive genomic profiling was performed using the FDA approved FoundationOne CDx assay (Foundation Medicine, Cambridge, MA) in a CLIA-certified, CAP-accredited laboratory, as previously described.^23^ FoundationOne CDx uses a hybrid capture methodology combined with bioinformatics to detect base substitutions, insertions/deletions, and copy number alterations in 324 genes and select gene rearrangements in 36 genes, as well as TMB and MSI. A board-certified pathologist reviewed a hematoxylin and eosin (H&E) slide from each sample under light microscopy to determine tumor adequacy (at least 20% tumor nuclei present) and review diagnosis. After CGP, a board-certified pathologist reviewed and approved the report. Tumor mutational burden was determined on a standard 0.79 Mb of sequenced DNA and assessment of MSI was performed by analysis of DNA sequencing across 114 loci as previously described.^24,25^ High TMB (TMB-H) was defined as ≥10 mutations/Mb. *CD274* gene copy number (CN) gain was defined as a *CD274* CN of at least one above the overall ploidy of the tumor specimen, copy number loss as a *CD274* CN at least one below the overall ploidy, and amplification as a *CD274* CN at least 4 above overall tumor ploidy. Predominant genetic ancestry of each patient was determined using principal component analysis of single-nucleotide polymorphisms based on their known variation amongst 5 ancestral superpopulations in the 1000 Genomes Project: African, Central and South American, East Asian, European, and South Asian.^26-28^ Ultraviolet (UV) mutational signatures were defined as described by Zehir et al^29^

### Statistical Analyses

We compared characteristics between our HPMel and LPMel cohorts, using Fisher’s exact test for categorical data, owing to the sizes of the cohorts, and the non-parametric Wilcoxon test for continuous parameters. A 2-tailed *P*-value of <.05 was considered to be statistically significant.

## Results

### Clinicopathologic Features of Patient Cohort

A total of 1268 consecutive patients with melanoma were tested for both PD-L1 DAKO 22C3 CDx IHC assay and CGP. The median and mean age of the cohort was 67 years old and 65.3 years old, with a female prevalence of 38.2% (484/1268). The majority of the patients were of European genetic ancestry (93.5%, 1186/1268) with separate additional subsets of Central and South American (4.4%, 56/1268), African (1.0%, 13/1268), East Asian (0.9%, 12/1268), and South Asian (0.1%, 1/1268) ancestry. Most of the specimens were from metastatic sites (69.6%, 883/1268) and a UV mutational signature was detected in approximately half of the cases (53.2%, 675/1268).

In total, 13.0% (165/1268) were HPMel and 87.01% (1103/1268) were LPMel. No significant differences were discovered in age, sex, genetic ancestry, primary vs metastatic sequenced site, and UV mutational signature between the HPMel and LPMel cohorts (*P* > .05) (Table 1).

**Table 1.** Clinicopathologic characteristics in heavily pigmented and non/lightly pigmented melanoma

**Clinicopathologic**	**Heavily pigmented melanoma (*n* = 165)**	***n***	**Non/lightly pigmented melanoma (*n* = 1103)**	***n***	***P*-value**
Age^a^					.26
Median	69		66		
Mean	66.5		65.1		
Female	33.9%	56	38.8%	428	.264
Predominant genetic ancestry					
African	2.4%	4	0.8%	9	.384
Central and South American	5.5%	9	4.3%	47	1
East Asian	1.2%	2	0.9%	10	1
European	90.3%	149	94.0%	1037	.437
South Asian	0.6%	1	0.0%	0	.651
Primary	29.7%	49	30.5%	336	.928
UV mutational signature	51.5%	85	53.5%	590	.676

Wilcox test, all other Fisher’s exact test. Predominant ancestry corrected for multiple comparison.

### Immunotherapy Biomarkers

Predictive ICPI biomarkers evaluated in the overall cohort (*n* = 1268 included TMB-H (54.5% positive, 691 patient case), MSI-H (0.1%, 1), *CD274* amplification (0.7%, 9), *CD274* CN gain (9.2%, 117), *CD274* CN loss (54.2%, 687), *PBRM1* GA (1.9%, 24), *STK11* GA (1.3%, 16), *KEAP1* GA (0.2%, 2), *MDM2* amplification (2.1%, 27), *MDM4* amplification (0.6%, 7), and APOBEC mutational signature (0.3%, 4).

Here, we saw a significantly reduced TMB (median 8.8 vs 11.4 mut/Mb, *P* = .007) and TMB-High status (47.3% [79/165] vs 54.6 [612/1103], *P* = .012] in the HPMel cohort when compared with the LPMel cohort. In addition, significantly higher prevalences of *CD274* CN gain (15.2% [25/165] vs 8.3% [92/1103], *P* = .015] and *STK11* GA (4.2% [7/165] vs 0.8% [9/1103], *P* = .005) were observed in HPMel cohort. When we examined the UV HPMel cohort and compared it with the UV LPMel cohort, the trends stayed the same (Supplementary Table S2). Otherwise, there was no significant difference between the HPMel and LPMel cohorts in the ICPI biomarkers examined in this study (*P* > .05; Table 2).

**Table 2.** Immunotherapy biomarker prevalence in heavily pigmented and non/lightly pigmented melanoma

Immunotherapy biomarkers	**Heavily pigmented melanoma (*n* = 165)**	***n***	**Non/lightly pigmented melanoma (*n* = 1103)**	***n***	***P*-value**
TMB					
Median	8.8		11.3		^a^.007
Mean	18.4		25.1		
TMB-H	47.3%	79	54.6%	612	.012
MSI-H	0.0%	0	0.1%	1	1
*CD274*					
Amplification	0.6%	1	0.7%	8	1
Copy number gain	15.2%	25	8.3%	92	.015
Copy number loss	44.8%	74	55.6%	613	.078
*PBRM1* GA	1.2%	2	2.0%	22	.561
*STK11* GA	4.2%	7	0.8%	9	.005
*KEAP1* GA	0.0%	0	0.2%	2	1
*MDM2*/4	*0.0%*		*0.0%*		
*MDM2* amplification	1.2%	2	2.3%	25	.410
*MDM4* amplification	0.0%	0	0.6%	7	.603
APOBEC mutational signature	0.0%	0	0.4%	4	1

Wilcox test; remaining evaluated with Fisher’s exact test.

**Figure 1.**
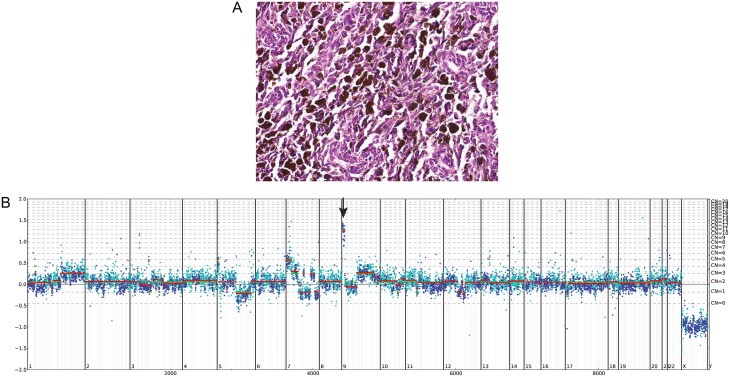
(**A**) Hematoxylin and Eosin (20× magnification) stained slide of a sample for which PD-L1 IHC was not evaluable as a result of heavy pigmentation. This melanoma case was from an 83-year-old male patient and was TMB-High (47 muts/Mb), MSS, and contained several genomic alterations including *APC* 904C>T; *PTCH1* 1930C>T; *TERTp* −124C>T; *TP53* 529C>T; *NF1* 3652C>T; *NF1* 5287C>T; *SETD2* 4168_4169insT. (**B**) Copy number plot that displays *CD274* amplification (CN: 11/ploidy + 9). This result was from sequencing of a melanoma from a 71-year-old male, and the sample was not evaluable for PD-L1 IHC due to melanin pigmentation. The tumor was not TMB-High and contained several genomic alterations including: *CDKN2A* 262G>T; *NRAS* 182A>G, *JAK2* amplification, *PDCD1LG2* amplification, and *ASXL1* deletion.

Importantly, in the HPMel cohort (*n* = 165), we saw similar frequency of MSI-H status (0%, 0), *CD274* amplification (0.6%, 1), *CD274* CN loss (44.8%, 74), *PBRM1* GA (1.2%, 2), *KEAP1* GA (0%, 0), *MDM2* amplification (1.2%, 2), *MDM4* amplification (0%, 0), and APOBEC mutational signature (0%, 0). An exemplary patient case was that of an 83-year-old male whose lymph node biopsy for melanoma was not evaluable for PD-L1 due to heavy pigmentation (Fig. 1A). Sequencing of the patient’s melanoma revealed TMB-High (47 muts/Mb) status, a predictive biomarker of response to ICPI. The tumor was microsatellite stable (MSS) and contained GA in *APC* 904C>T; *PTCH1* 1930C>T; *TERT*p −124C>T; *TP53* 529C>T; *NF1* 3652C>T; *NF1* 5287C>T; *SETD2* 4168_4169insT. Another melanoma sample from a 71-year-old male was also not evaluable for PD-L1 IHC due to melanin pigmentation. This case showed *CD274* amplification (CN: 11/ploidy + 9), implying likely overexpression of PD-L1 if the specimen was evaluable by IHC (Fig. 1B). This patient sample was not TMB-High, was MSS, and displayed several additional GA including: *CDKN2A* 262G>T; *NRAS* 182A>G, *JAK2* amplification, *PDCD1LG2* amplification, and *ASXL1* deletion.

### Genomic Differences Between HPMel and LPMel

In the overall melanoma cohort (*n* = 1268), GA were present in *TERTp* (65.9%, 835), *TP53* (23.9%, 303), *CDKN2A* (44.7%, 567), *BRAF* (41.0%, 520), *NRAS* (22.6%, 287), *CTNNB1* (4.7%, 60), *PRKAR1A* (0.9%, 11), *GNAQ* (3.5%, 45), *GNA11* (2.7%, 34), *KIT* (5.8%, 74), *PTEN* (15.3%, 194), and *BAP1* (4.7%, 59). No significant difference in *BRAF* V600E mutations was observed between the HPMel and LPMel cohort (18.2% [30/165] vs 25.4% [280/1103], *P* = .052).

**Figure 2.**
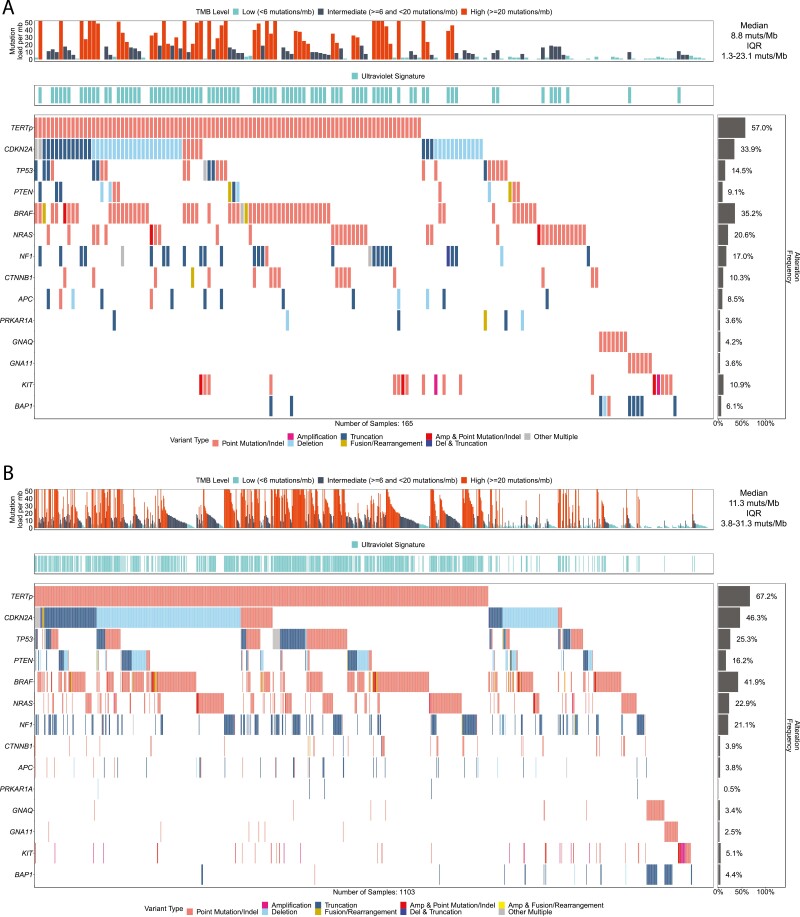
(**A**) Co-mutation plots of the 14 most clinically relevant genes in melanomas of the HPMel and (**B**) LPMel cohorts. Of these 14 genes, 8 genes had significantly different frequencies of GA between the HPMel and LPMel, suggesting a different genomic profile between these cohorts. The frequency of GA in *TERTp* (67.2% [741/1103] vs 57.0% [94/165]), *TP53* (25.3% [279/1103] vs 14.5% [24/165]), *CDKN2A* (46.3% [511/1103] vs 33.9% [56/165]), and *PTEN* (16.2% [179/1103] vs 9.1% [15/165]) was significantly higher in the LPMel cases (*P* = .011, .002, .003, .020, respectively). A higher rate of GA in *CTNNB1* (10.3% [17/165] vs 3.9% [43/1103]), *APC* (8.5% [14/165] vs 3.8% [42/1103]), *PRKAR1A* (3.6% [6/165] vs. 0.5% [5/1103]), and *KIT* (10.9% [18/165] vs 5.1% [56/1103]) was identified in the HPMel cohort (*P* = .001, .013, .001, .006, respectively).

Co-mutation plots of the 14 most clinically relevant genes in melanomas of the HPMel and LPMel cohorts are shown in Fig. 2A and B. Of these 14 genes, 8 genes had significantly different frequencies of GA between the HPMel and LPMel, suggesting a different genomic profile of these cohorts (Table 3). The frequency of GA in *TERTp* (67.2% [741/1103] vs 57.0% [94/165]), *TP53* (25.3% [279/1103] vs 14.5% [24/165]), *CDKN2A* (46.3% [511/1103] vs 33.9% [56/165]), and *PTEN* (16.2% [179/1103] vs 9.1% [15/165]) was significantly higher in the LPMel cases (*P* = .011, .002, .003, .020, respectively). A higher rate of GA in *CTNNB1* (10.3% [17/165] vs 3.9% [43/1103]), *APC* (8.5% [14/165] vs 3.8% [42/1103]), *PRKAR1A* (3.6% [6/165] vs. 0.5% [5/1103]), and *KIT* (10.9% [18/165] vs 5.1% [56/1103]) was identified in the HPMel cohort (*P* = .001, .013, .001, .006, respectively). Of note, 8/18 of the *KIT-*mutant HPMel cases were anogenital mucosa primary and 3/18 were acral primary. When we examined the UV HPMel cohort and compared it with the UV LPMel cohort these trends stayed the same (Supplementary Table S2).

**Table 3.** Genomic profile in heavily pigmented and non/lightly pigmented melanoma

**Genes**	**Heavily pigmented melanoma (*n* = 165)**	***n***	**Non/lightly pigmented melanoma (*n* = 1103)**	***n***	***P*-value** ^a^
*TERTp*	57.0%	94	67.2%	741	.011
*CDKN2A*	33.9%	56	46.3%	511	.003
*TP53*	14.5%	24	25.3%	279	.002
*PTEN*	9.1%	15	16.2%	179	.020
*BRAF*	35.2%	58	41.9%	462	.107
*V600E*	18.2%	30	25.4%	280	.052
*non-V600E*	17.0%	28	16.5%	182	.910
*NRAS*	20.6%	34	22.9%	253	.550
*NF1*	17.0%	28	21.1%	233	.256
*CTNNB1*	10.3%	17	3.9%	43	.001
*APC*	8.5%	14	3.8%	42	.013
*PRKAR1A*	3.6%	6	0.5%	5	.001
*GNAQ*	4.2%	7	3.4%	38	.650
*GNA11*	3.6%	6	2.5%	28	.434
*KIT*	10.9%	18	5.1%	56	.006
*BAP1*	6.1%	10	4.4%	49	.326

All Comparisons Performed with Fisher’s exact test.

## Discussion

In this study, we have shown that less pigmented melanomas harbor more frequent GAs in *TERTp*, *CDKN2A*, *TP53*, and *PTEN*, mutations that are classic in the progression of melanomas. These results suggest that LPMel have progressed further along this genomic pathway than HPMel.^30^ This, along with the lower TMB and TMB-H status in the HPMel, suggests that pigmented melanomas in general are molecularly less developed and may have a reduced response to ICPI. However, there is marked overlap in TMB between the 2 groups, suggesting that HPMel would nevertheless benefit from ICPI.

Melanomas in sun damaged skin are divided in the current World Health Organization Classification into low cumulative sun damage (CSD) and high CSD, based on the degree of solar elastosis in background dermis, with resulting clinicopathologic and genomic differences.^31^ Crucial genomic differences include the identification of *BRAF* V600E alterations in low CSD melanoma and by contrast *BRAF* non-V600E and *NF1* alterations in high CSD melanoma. A limitation of this study was that stratification into these 2 CSD types was not possible as background solar elastosis was not evaluable for the majority of cases. However, the frequency of GAs in *BRAF* V600E, *BRAF* non-V600E, and *NF1* did not show significant differences between the HPMel and LPMel groups (Table 3), indicating that it is likely that both groups contain similar proportions of low and high CSD tumors.

An enrichment of GA in *CTNNB1/APC* and *PRKAR1A* was identified in the HPMel cohort. This is consistent with the literature that GA in these genes are associated with heavily pigmented melanocytic tumors. Specifically, it is well-established that GAs in *CTNNB1/APC* and *PRKAR1A* are associated with deep penetrating nevus and pigmented epithelioid melanocytoma, respectively, both of which have abundant melanin pigment as a histolopathologic feature.^32-35^ These alterations have also been described in specific subsets of low CSD melanoma.^31^ The increased percentage of specifically *KIT* alterations in the HPMel group is likely secondary to enrichment for anogenital mucosal primary site cases (8/18 cases), which often contain increased pigmentation.^36,37^

From a practical clinical standpoint, this is the first study in the literature that presents real-world failure rates of PD-L1 IHC in HPMel. These cases account for 13.0% (165/1268) of the total population of melanomas submitted for testing at our institution. Almost half were TMB-H, a predictive biomarker that these patients with TMB-H HPMel are likely to respond to ICPI. In addition to the presence of other positive predictive biomarkers such as *CD274* amplification and CN gains, negative predictive biomarkers such as *CD274* CN loss, *STK11* GA, and *MDM2* amplification were also present. These findings are further exemplified by the 2 patient cases presented in this study where we saw one patient with HPMel with high TMB and one patient with HPMel with *CD274* amplification. Given that a high percentage of these melanoma cases that fail PD-L1 IHC show other biomarkers predictive of response to ICPI, it appears important to test HPMel with CGP in order to guide treatment decisions.

With respect to limitations, it is unclear whether the HPMel cases were sent because the samples were the best available or if the sending physicians were unaware of the complications with samples with extensive pigmentation. Further education in best samples to submit for both PD-L1 IHC and CGP is warranted and may lower real-world failure rates of PD-L1 IHC. Additional studies on the potential utility of red chromogen, such as Fast Red, for PD-L1 IHC may also be warranted.^38^ Another limitation of this study is that the cases included were collected from patients with advanced malignancies, submitted for detection of targetable biomarkers and GA. Thus, these cases likely exemplify the aggressive end of the biologic spectrum without representing early, thin, and/or indolent melanomas. Finally, follow-up data were not available in this study, but will be important to obtain for future studies of HPMel to correlate with therapeutic outcomes.

## Conclusions

In this study, we quantified the failure rates of melanoma samples for PD-L1 testing due to high melanin pigmentation and showed that CGP can be used in these patients to identify biomarkers that can guide treatment decisions for patients with HPMel. We show that pigmented melanomas in general are molecularly less developed, with a significantly lower but highly overlapping TMB. Additional studies are needed to evaluate if extensive pigmentation in melanoma may be an independent negative predictor for response to immunotherapy.

## Supplementary Material

oyac090_suppl_Supplementary_Material

## Data Availability

The data underlying this article are available in the article and in its online supplementary material.
