# Supplementary material for: Variable Genomic Landscapes of Advanced Melanomas with Heavy Pigmentation
Source: Oncologist. 2022 May 12;27(8):655–62. doi: 10.1093/oncolo/oyac090 (PMC9355815; doi:10.1093/oncolo/oyac090)
Supplement: oyac090_suppl_Supplementary_Material [file oyac090_suppl_supplementary_material.docx]

**Supplement**

**Supplemental Table 1**

| **Immunotherapy Biomarkers** | **UV Heavily pigmented melanoma (n=85)** | **n** | **UV Non/lightly pigmented melanoma (n=590)** | **n** | **p-value** |
| --- | --- | --- | --- | --- | --- |
| **TMB** |  |  |  |  |  |
| *median* | 21.3 |  | 27.5 |  | *0.088 |
| *mean* | 33.2 |  | 42.2 |  |  |
| *TMB-H* | 88.2% | 75 | 93.4% | 551 | 0.113 |
| **MSI-H** | 0.0% | 0 | 0.0% | 0 | 1 |
| ***CD274*** |  |  |  |  |  |
| *amplification* | 0.0% | 0 | 0.8% | 5 | 1 |
| *copy number gain* | 7.1% | 6 | 9.2% | 54 | 0.684 |
| *copy number loss* | 49.4% | 42 | 58.0% | 342 | 0.160 |
| ***PBRM1* GA** | 2.4% | 2 | 2.5% | 15 | 1 |
| ***STK11* GA** | 3.5% | 3 | 1.2% | 7 | 0.120 |
| ***KEAP1* GA** | 0.0% | 0 | 0.3% | 2 | 1 |
| ***MDM2/4*** |  |  |  |  |  |
| *MDM2 amplification* | 1.2% | 1 | 0.7% | 4 | 0.491 |
| *MDM4 amplification* | 0.0% | 0 | 0.3% | 2 | 1 |

*Wilcox test; remaining evaluated with Fisher’s exact test

**Supplemental Table 2**

| **Genes** | **UV Heavily pigmented melanoma (n=85)** | **n** | **UV Non/lightly pigmented melanoma (n=590)** | **n** | **p-value** |
| --- | --- | --- | --- | --- | --- |
| *TERTp* | 83.5% | 71 | 89.8% | 530 | 0.094 |
| *CDKN2A* | 43.5% | 37 | 48.5% | 286 | 0.418 |
| *TP53* | 16.5% | 14 | 36.8% | 217 | <0.001 |
| *PTEN* | 9.4% | 8 | 16.4% | 97 | 0.109 |
| *BRAF* | 43.5% | 37 | 41.7% | 246 | 0.814 |
| *V600E* | 16.5% | 14 | 19.8% | 117 | 0.558 |
| *non-V600E* | 27.1% | 23 | 21.9% | 129 | 0.330 |
| *NRAS* | 25.9% | 22 | 27.5% | 162 | 0.796 |
| *NF1* | 28.2% | 24 | 31.0% | 183 | 0.706 |
| *CTNNB1* | 12.9% | 11 | 4.6% | 27 | 0.004 |
| *APC* | 9.4% | 8 | 5.8% | 34 | 0.225 |
| *PRKAR1A* | 2.4% | 2 | 0.7% | 4 | 0.168 |
| *GNAQ* | 0% | 0 | 0.7% | 4 | 1 |
| *GNA11* | 1.2% | 1 | 1.2% | 7 | 1 |
| *KIT* | 5.9% | 5 | 2.5% | 15 | 0.094 |
| *BAP1* | 3.5% | 3 | 0.5% | 3 | 0.029 |

Fisher’s exact test
